# *TempEasy* 3D Hydrogel Coculture System Provides Mechanistic Insights into Prostate Cancer Bone Metastasis

**DOI:** 10.1021/acsami.4c03453

**Published:** 2024-05-13

**Authors:** Zhaobao Zhang, Wen Chen, Mingchen Sun, Tilly Aalders, Gerald W. Verhaegh, Paul H. J. Kouwer

**Affiliations:** † Institute for Molecules and Materials, Radboud University, Heyendaalseweg 135, Nijmegen 6525 AJ, The Netherlands; ‡ Department of Urology, Radboud Institute for Molecular Life Sciences, Radboud university medical center, Geert Grooteplein Zuid 28, Nijmegen 6525 GA, The Netherlands

**Keywords:** 3D cocultures, polyisocyanide hydrogels, artificial extracellular matrices, prostate cancer, bone metastasis, proliferation, neovascularization, paracrine cell−cell signaling

## Abstract

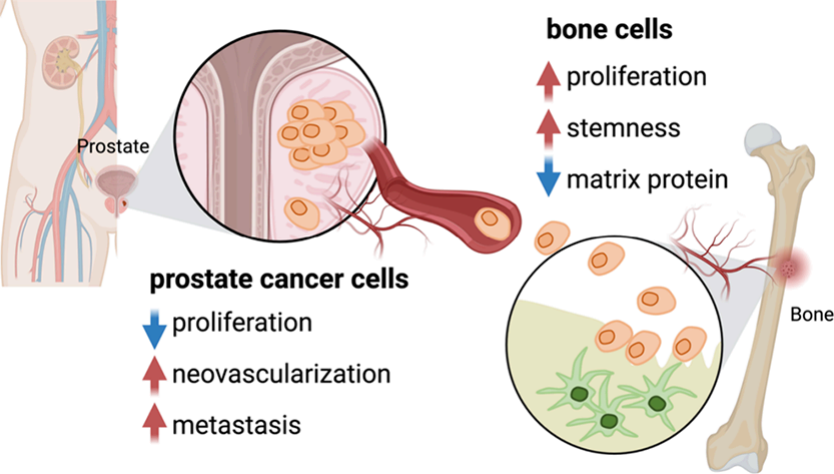

Patients diagnosed with advanced prostate cancer (PCa) often experience incurable bone metastases; however, a lack of relevant experimental models has hampered the study of disease mechanisms and the development of therapeutic strategies. In this study, we employed the recently established Temperature-based Easy-separable (*TempEasy*) 3D cell coculture system to investigate PCa bone metastasis. Through coculturing PCa and bone cells for 7 days, our results showed a reduction in PCa cell proliferation, an increase in neovascularization, and an enhanced metastasis potential when cocultured with bone cells. Additionally, we observed increased cell proliferation, higher stemness, and decreased bone matrix protein expression in bone cells when cocultured with PCa cells. Furthermore, we demonstrated that the stiffness of the extracellular matrix had a negligible impact on molecular responses in both primary (PCa cells) and distant malignant (bone cells) sites. The *TempEasy* 3D hydrogel coculture system is an easy-to-use and versatile coculture system that provides valuable insights into the mechanisms of cell–cell communication and interaction in cancer metastasis.

## 1 Introduction

In 2018, nearly 1.2 million new cases were projected to be diagnosed with prostate cancer (PCa),^1^ and its incidence is increasing steadily.^2,3^ Patients with advanced PCa frequently develop bone metastases that are incurable.^4,5^ Given that bone metastasis is the main cause of mortality, there is an urgent need in understanding the nuances of the organotrophic spread, to study tumorigenesis, and to benefit drug discovery.

Several preclinical models, developed so far, have elucidated the pathophysiology of PCa, but the field is hampered by the lack of relevant models.^6^ Cell culture is a fundamental technique used to study PCa biology, and two-dimensional (2D) cell cultures are mostly used for *in vitro* studies. PCa cell lines solely grown in 2D cultures, however fail to recapitulate PCa cells derived from patient tumors, showing fundamental differences in cell morphology, proliferation, and cellular signaling pathways.^7,8^ This shortcoming is one of the main reasons why the US National Cancer Institute (NCI) has decided to retire its panel of 60 human cancer cell lines grown in monoculture from a drug-screening program but refocus on developing “patient-derived xenografts” (PDXs).^9^ Such models promise to capture the genetic complexity of cancers, but PDXs also have shortcomings.^10^ Its development usually requires more than 6 months, and such delay limits the utility of PDX models in immediate patient treatment. Additionally, it is difficult to establish its PDX models for PCa, because not all factors needed for proper tumor growth are known,^10,11^ while others produce metastases that primarily localize to the wrong organs, such as the lymph nodes or lung, and only sporadically develop bone metastases.^6^

To overcome the limitations mentioned above, three-dimensional (3D) cell culture methods have been developed that bridge the gap between the conventional 2D cultures and mouse models.^12^ Studies have shown that PCa cells cultured in extracellular matrix-like gels often spontaneously aggregate, which gives rise to the formation of tumoroids that not only display cell–cell adhesions but also cell-extracellular matrix interactions.^13−15^ To support PCa growth and phenotypic expression that mimic different stages of metastatic progression, 3D scaffolds with adjustable scaffold stiffness have been developed.^16−18^ Besides necessary interactions with the 3D microenvironment, interactions between different cells are key components that drive PCa progression. These effects have been largely ignored in previous experimental setups. We note that bone is often the only clinically detectable site of metastasis, and the resulting tumors tend to be osteoblastic rather than osteolytic.^4^ Beyond question, communication between bone and PCa cells is crucial in promoting the development of bone metastases.^19^ So far, few studies investigated this key aspect in 3D settings.^20−22^

Earlier, we developed TempEasy,^23^ a user-friendly indirect 3D coculture system. This system employs the synthetic oligo(ethylene glycol)-substituted polyisocyanide (PIC) gels.^24,25^ Mounting experimental evidence from a wide range of *in vitro* cell culture^26^ and organoid^27−29^ studies as well as *in vivo* animal experiments^30−32^ support the outstanding biocompatibility of PIC hydrogel. *TempEasy* capitalizes on the thermoreversible gelation of the PIC gels. It comprises two distinct PIC gels with adjustable stiffness and different gelation temperatures (*T*_gel_). The thermo-reversibility of *TempEasy* simplifies cell extraction and allows for downstream analysis.

Here, we use *TempEasy* to study the underlying mechanisms involved in the preferential colonization of disseminated PCa cells in bone tissue. To this end, we culture MSK-PCa1 prostate cancer cells with MG-63 bone cells in the indirect 3D *TempEasy* coculture platform, which allows us to study direct paracrine effects. In addition, we examine the influence of extracellular matrix (ECM) stiffness on PCa cells at the primary site and bone cells at distant metastatic sites. This investigation is particularly significant given the limited understanding of the relationship between ECM mechanical properties and the malignant potential of advanced PCa.^33^ While previous 3D coculture studies in PCa primarily focused on establishing *in vitro* models,^17,20,34^ our study provides valuable insights into the intricate mechanisms that govern PCa bone metastasis.

## 2 Results

### 2.1 Setup and Analysis of the *TempEasy* 3D Coculture System to Study PCa Bone Metastasis

**Figure 1:**
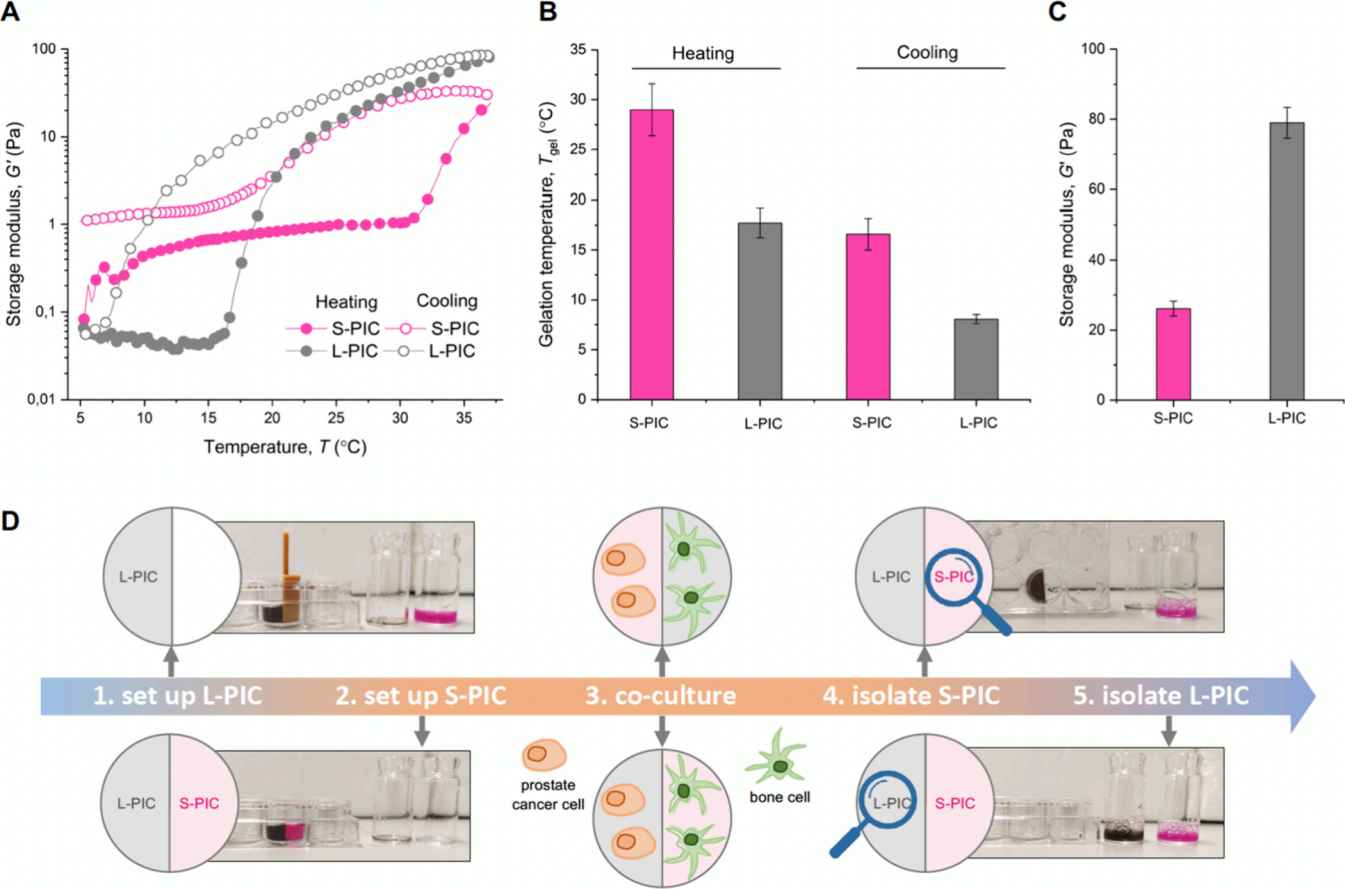
*TempEasy* 3D coculture system for PCa/bone cell cocultures. (A) The temperature-responsive storage shear modulus *G*′ was measured for both **L-PIC** (gray) and **S-PIC** (pink), each at a 1.5 mg/mL concentration. (B) Gelation temperatures *T*_gel_ of **L-PIC** and **S-PIC** hydrogels during heating and cooling. (C) Storage modulus *G*′ of **L-PIC** and **S-PIC** at 37 °C. (D) Photos to illustrate thermo-reversibility of the *TempEasy* 3D coculture system: **S-PIC** (1.5 mg/mL) was dissolved in a medium (pink), and **L-PIC** (1.5 mg/mL) was dissolved in a medium with a black dye to visualize the two compartments. At room temperature (∼20 °C), **L-PIC** solidifies into a hydrogel, and the inset is removed. Once combined with **S-PIC** and heated to 37 °C, a two-compartment coculture system is realized. Upon cooling to 17 °C, the **S-PIC** compartment liquifies and can be isolated without disturbing the **L-PIC** compartment, which remains a gel until further cooling to 4 °C for extraction. It is noteworthy that there is no visible contamination between the **L-PIC** and **S-PIC** compartments.

The versatility of polyisocyanide (PIC) gel as a synthetic material lies in its tailorable structure and mechanical properties.^35^ In this study, we synthesized PIC polymers incorporating a small proportion (3.3 mol %) of an azide-functionalized monomer for subsequent modification, similar to a previous study.^36^ We prepared two polymer variants with different chain lengths (*L*_C_) by varying the monomer-to-catalyst ratio during polymerization, which resulted in a “short” polymer, labeled **S-PIC**-N3 (*L*_C_ = 99 nm and a viscosity average molecular weight, *M*_v_ = 251 kg/mol) and a “long” polymer, labeled as **L-PIC**-N3 (*L*_C_ = 222 nm, *M*_v_ = 562 kg/mol). To introduce cell-matrix interactions, we conjugated the cell-binding peptide GRGDS, linked to a DBCO-terminated PEG spacer, to the azide group on the polymers, resulting in target polymers **S-PIC** and **L-PIC**. Based on the high conversion rates in the literature, we estimate a peptide density of ∼3% per monomer.^37,38^ Gels were prepared by dissolving the polymers in the medium at 4 °C before warming the solutions to 37 °C. To assess the mechanical properties of the gels, oscillatory shear rheology was employed. Gels of **S-PIC** and **L-PIC** displayed distinct gelation temperatures (*T*_gel_), clearly marked by the increase in the shear modulus at their respective *T*_gel_ (Figure 1A). Specifically, the *T*_gel_ of **S-PIC** was ∼30 °C, and **L-PIC** exhibited a *T*_gel_ of ∼18 °C (Figure 1B). Upon cooling, both **S-PIC** and **L-PIC** exhibited gel-to-sol transitions, with **S-PIC** undergoing the transition at 17 °C and **L-PIC** at 8 °C (Figure 1B). In addition, the storage modulus *G′* of **S-PIC** and **L-PIC** is 26 ± 2 and 79 ± 4 Pa, respectively, indicating that **L-PIC** is stiffer than **S-PIC** (Figure 1C and Figure S1).

The different *T*_gel_ values of **S-PIC** and **L-PIC** allow for a selective separation by precisely controlling the temperature (Figure 1D). In a medium, both materials form elastic gels at 37 °C. When the temperature is reduced from 37 to 15 °C, **S-PIC** reverts to a fluid state with low viscosity, enabling its selective removal, while **L-PIC** remains in the gel state. Subsequent cooling to 4 °C allowed the extraction of the **L-PIC** gel. Thus, the unique *T*_gel_ of the polymers, as a result of their different molecular weights, serves as the foundation for our *TempEasy* 3D cell coculture system.

### 2.2 Cell Morphologies and Gene Expression of PCa Cells in **S-PIC** of *TempEasy*

**Figure 2:**
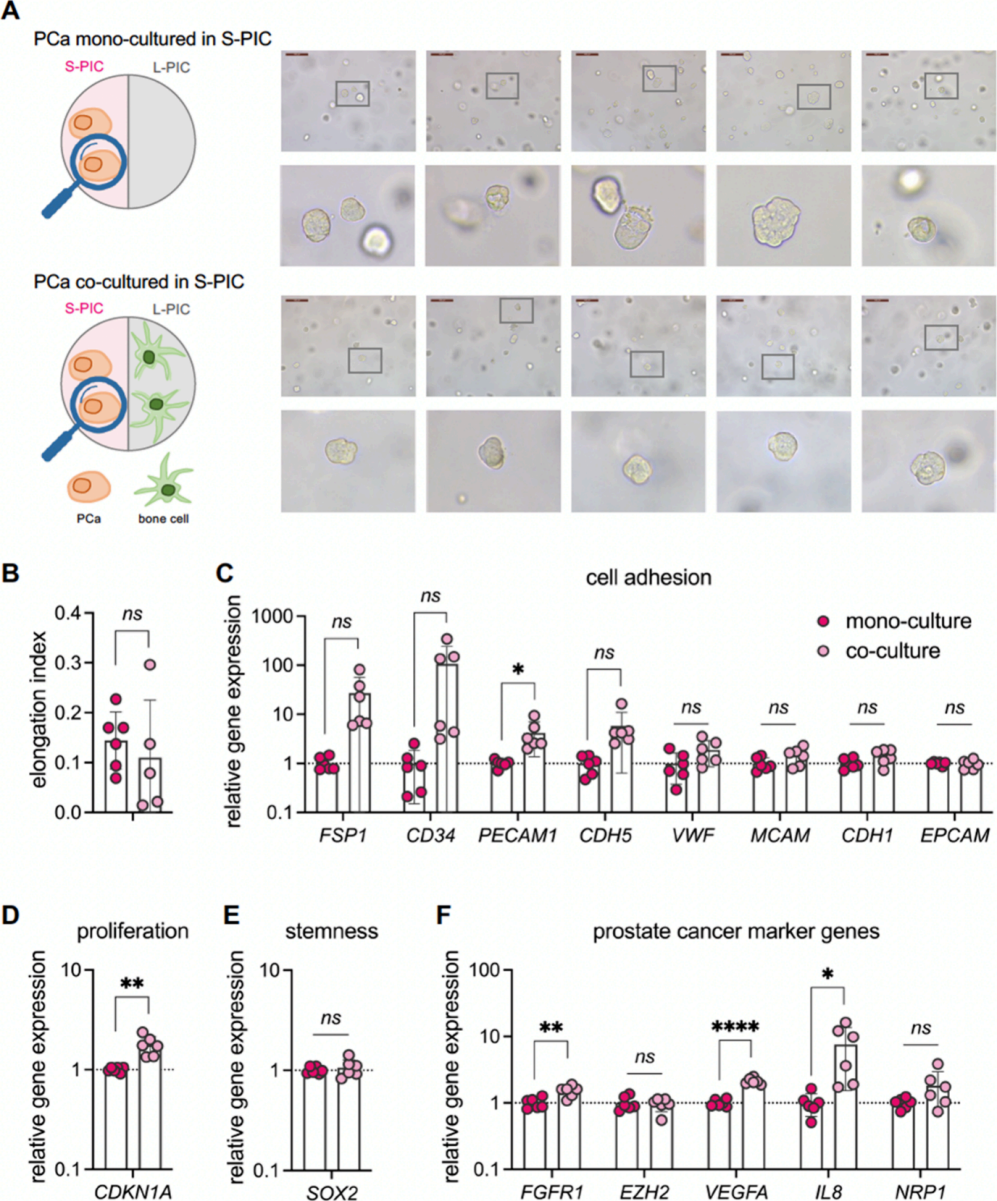
PCa cells in **S-PIC** of *TempEasy*. (A) Schematics of our experimental setup. Brightfield microscopy images of PCa cells after 7 days in **S-PIC** hydrogel, either monoculture (top panel) or coculture with bone cells (bottom panel). For cell culture details, see the Materials and Methods section. (B) Quantification of the elongation index of PCa cells. (C–F) RT-qPCR quantification of marker gene expression, including cell adhesion makers (C), cell proliferation (D), stemness (E), and reported PCa progression markers (F), by comparing monoculture to coculture with bone cells. Human acidic ribosomal protein (*hARP*) served as the normalization reference gene. The expression differences were assessed using the 2ΔΔ*C*_t_ method.^44^ Primer sequences utilized for qPCR are listed in Table 1. The results are expressed as mean ± SD of six biological replicates. Statistical significance was determined using an unpaired *t* test with Welch’s correction: *ns* = not significant (*p* >0.05), *p <0.05, **p <0.01, ****p <0.0001.

In the process of bone metastasis, the PCa cell secretome will affect the bone cells, and vice versa. In the next sections, we study both contributions systematically. In earlier work, it was shown that PCa tissues, especially in advanced PCa, were softer than that of the noncancerous benign prostatic hyperplasia tissues on the microscale.^39^ Therefore, we first seeded PCa cells in the softer **S-PIC** hydrogel and we compared cell morphologies and gene expression of the PCa cells in the coculture with the bone cells in the stiffer L-PIC to the PCa cells in monoculture (Figure 2A). After a 7-day monoculture or coculture with bone cells, PCa cells in the **S-PIC** hydrogel retained their rounded shape. The results are quantified by the elongation index, which describes the extent of the equimomental ellipse,^40,41^ which confirms that 3D coculture with bone cells introduces no significant morphological change in PCa cells (Figure 2B).

As not all cell–cell interactions and responsiveness to the microenvironment can be reflected by morphological changes, we explored gene expression with RT-qPCR (Figure 2C–F). We evaluated changes in the expression of genes involved in a range of key processes and characteristics, including cell adhesion (*FSP1*, *CD34*, *PECAM1*, *CDH5*, *VWF*, *MCAM*, *CDH1*, and *EPCAM*), cell proliferation (*CDKN1A*), stemness (*SOX2*), and reported PCa progression markers (*FGFR1*, *EZH2*, *VEGFA*, *IL8*, and *NRP1*).

In terms of cell adhesion markers, we observed a significantly upregulated *PECAM1* expression in PCa when cocultured with bone cells (Figure 2C). *PECAM1* (Platelet Endothelial Cell Adhesion Molecule 1) or *CD31* is mainly involved in the regulation of cell–cell interactions and angiogenesis.^42^ A previous study identified that the expression of *PECAM1* is associated with the early angiogenic switch and the recruitment of new vasculature to lesions indicative of advanced prostatic epithelial neoplasia,^43^ which highlights the important role that *PECAM1* plays in the development of new blood vessels during the progression of PCa.

In addition, we observed a similar upregulation in the expression of *FSP1*, *CD34*, and *CDH5*, when cocultured with bone cells; however, the difference is not significant between monoculture and coculture (Figure 2C). *FSP1*, also known as *S100A4* (calcium-binding protein S100A4), is known to accelerate tumorigenesis as well as invasion of human prostate cancer via the transcriptional control of matrix metalloproteinase 9.^45,46^*CD34* is a surface marker found on hematopoietic stem cells and is observed to be frequently expressed on the vascular endothelium of newly formed blood vessels. Previous studies showed that increased expression of *CD34* confers tumor progression and aggressiveness in prostate cancer,^47^ with a high expression of *CD34* in tumor tissue that suggests intensive tumor neovascularization.^48^ Expression of *CDH5*, encoding for VE-Cadherin, another glycoprotein important for cell–cell adhesion, signal transduction, and for vascular remodeling^49^ is also higher in PCa cells when cocultured with bone cells (Figure 2C). Importantly, a recent study showed that aberrant extravascular expression of *CDH5* has been observed in certain cancer types associated with vasculogenic mimicry, which is a blood supply system separate from endothelial vessels within tumor cells. This mechanism underlines the large adaptability of aggressive tumor cells that express vascular cell markers and form structures resembling tumor vasculature.^50^ Thus, the higher expression of *PECAM1*, together with *CD34* and *CDH5*, likely suggested that the bone cells increase the neovascularization potential of PCa cells in the cocultures. This increased angiogenic potential may contribute to the growth and spread of PCa.

In contrast, there were no clear changes in the expression of *VWF*, *MCAM*, *CDH1*, and *EPCAM*, which have also been reported to be involved in carcinomas and their metastases (Figure 2C). Therefore, it is likely that coculture with bone cells specifically influences some cell adhesion markers in PCa while leaving other adhesion molecules unaffected.

The effect of the coculture on PCa cell proliferation was assessed with the cell cycle marker, *CDKN1A*, a broad-acting cyclin-dependent kinase inhibitor that encodes for the protein p21, whose expression anticorrelates with PCa growth *in vitro* and *in vivo*(51−53) (Figure 2D). We observed significantly higher *CDKN1A* expression in PCa cells when cocultured with bone cells, suggesting slower cell proliferation.

We also tested the stem cell marker SOX2, because in a large variety of different human cancers, it was found to be amplified or overexpressed. Enhanced SOX2 expression is considered to drive neoplastic progression by accelerating cancer migration, invasion, and metastasis. Furthermore, an increased *SOX2* expression is associated with increased drug resistance and poor survival of cancer patients.^54,55^ However, we did not observe any clear difference between monoculture and coculture with bone cells (Figure 2E).

Finally, we tested several marker genes that have been reported in advanced PCa, including *FGFR1*, *EZH2*, *VEGFA*, *IL8*, and *NRP1* (Figure 2F). Among these marker genes, we observed a significant increase in the expression of *FGFR1*, *VEGFA*, and *IL8*, but not EZH2 and *NRP1* (Figure 2F). *FGFR1* (Fibroblast Growth Factor Receptor 1) has been linked to the development and progression of various cancer types, including prostate cancer. The upregulated *FGFR1* expression in our model is in agreement with a recent study, where researchers showed that hydrogel-encapsulated PCa 118b cells expressed higher *FGFR1*, which is also highly expressed by these cells *in vivo*.^34^ Similarly, we observed an elevated expression of two important proangiogenic factors, *VEGFA* and *IL8*, which is in line with literature reports.^56^ Collectively, these findings suggested that overexpression of these markers in PCa cells likely originates from the presence of the bone cells.

The absence of a clear effect on *EZH2* is opposite to an earlier report that found that *EZH2* expression was significantly higher in metastatic prostate cancer compared to clinically localized prostate cancer, and in localized prostate cancer compared to benign prostate tissue.^57^ The same holds for the marker *NRP1* (Neuropilin 1),^58,59^ which overexpression usually correlates with tumor aggressiveness, metastasis, and poor prognosis.^60^ We did not observe any clear change in the expression of *NRP1* in PCa cells due to the presence of bone cells. These seemingly contradictory results may reflect that our current 7-day coculture setup fails to mimic all different stages of PCa bone metastasis, especially the late stage, considering the highly dynamic gene expression patterns during metastasis.

To sum up, in the *TempEasy* 3D coculture system, we did not observe any clear morphological changes of PCa cells upon coculturing with bone cells. However, gene expression analysis yielded major findings in PCa cells: (1) An increase in adhesion markers (*PECAM1*, *CD34*, *CDH5*) suggests that coculture with bone cells enhances the neovascularization potential of PCa cells, which may serve as a molecular mechanism underlying the preferential spreading of PCa to bone *in vivo*. (2) PCa cells proliferate less when cocultured with bone cells, which indicates that bone metastasis is probably not a result of enhanced PCa cell proliferation. (3) Elevated gene expression of metastasis markers (*FGFR1*, *VEGFA*, and *IL8*) in PCa cells is likely due to the cell–cell communication with bone cells via signaling molecules.

### 2.3 Cell Morphologies and Gene Expression of PCa Cells in **L-PIC** of *TempEasy*

**Figure 3:**
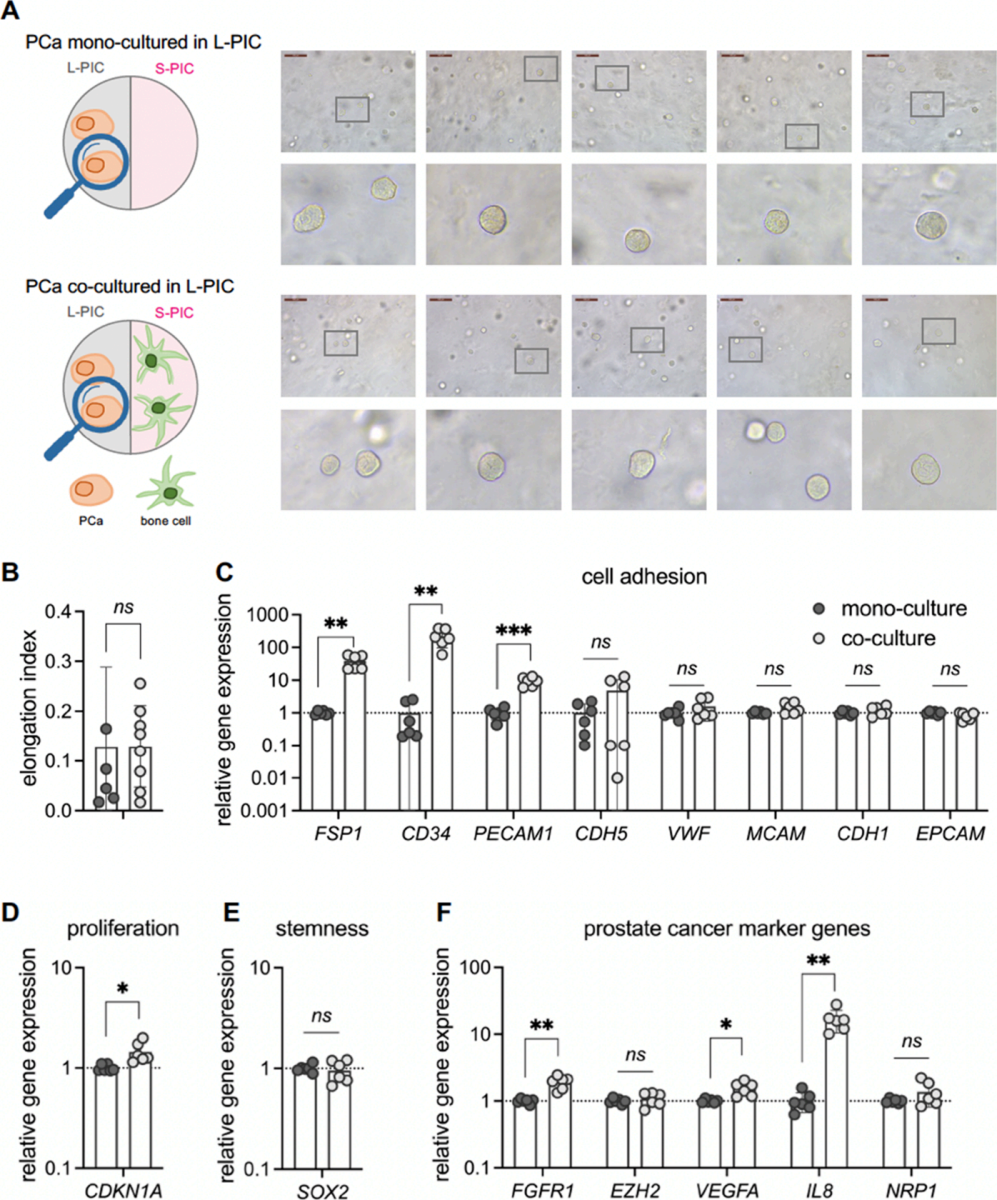
PCa cells in the **L-PIC** of *TempEasy*. (A) Schematics of our experimental setup. Brightfield microscopy images of PCa cells after 7 days in **L-PIC** hydrogel culture, either monoculture (top panel) or coculture with bone cells (bottom panel). (B) Quantification of elongation index of PCa cells. (C–F) RT-qPCR quantification of marker gene expression, including cell adhesion makers (C), cell proliferation (D), stemness (E), as well as reported PCa markers (F), by comparing monoculture to coculture with bone cells. Results are expressed as mean ± SD of six biological replicates. Statistical significance was determined using an unpaired *t* test with Welch’s correction: *ns* = not significant (*p* >0.05), *p <0.05, **p <0.01, ***p <0.001.

It is well-known that the tumor microenvironment is crucial for neoplastic cell initiation or tumorigenesis, progression, and metastasis of tumor cells.^61^ Studies from breast cancer revealed that human breast cancer transformation involves a gradual increase in collagen deposition and a continuous linearization and thickening of interstitial collagen.^62^ Hence, we further explored whether higher stiffness of the ECM would result in a different response. PCa cells were seeded in the stiffer **L-PIC** hydrogel, and we compared the monoculture to the coculture with the bone cells (Figure 3A). After 7-day monoculture or coculture with bone cells in **L-PIC** hydrogel, PCa cells displayed a rounded shape, similar to that observed in **S-PIC** hydrogel (Figure 2), as supported by the elongation index^40,41^ (Figure 3B).

Gene expression was analyzed using the same panel of genes as used before. In general, we observed consistent trends in all tested genes, with minor differences in significance (Figure 3C–F). For instance, compared with monoculture, PCa cells in **L-PIC** hydrogel when cocultured with bone cells showed a significantly higher expression *FSP1*, *CD34*, and *PECAM1*, whereas other cell adhesion markers were not affected (Figure 3C). Notably, the expression of *CD34* is over 100-fold higher when cocultured with bone cells, showing the strongest effect among tested markers. Given its role in tumor neovascularization,^48^*CD34* may serve as a promising target for developing new treatments. In addition, we observed a significantly higher *CDKN1A* expression, implying slower cell proliferation (Figure 3D). Besides, the expression of *SOX2* is unchanged (Figure 3E), which is also in line with that observed in the **S-PIC** hydrogel (Figure 2E). The same is true for previously reported PCa progression marker genes, where a significant increase in the expression of *FGFR1*, *VEGFA*, and *IL8* is observed, but not in *EZH2* and *NRP1* (Figure 3F).

Whether cultured in **S-PIC** (Figure 2) or in **L-PIC** (Figure 3), the PCa cells are strongly affected by the presence of the bone cells in the other compartment. Clearly, though, the **L-PIC** and **S-PIC** matrices differ in (mechanical) properties, which may also affect cell behavior. To exclude that differences in mechanics of both matrices play a significant role, we also compared gene expression of the PCa cells in **L-PIC** and **S-PIC** in the monoculture (Figure S2) and in the coculture (Figure S3). We find that virtually all expression levels are statistically the same; only the PCa expression of *VEGFA* in the stiffer **L-PIC** monoculture was slightly, but significantly, elevated compared to the **S-PIC** monoculture. For the coculture comparison, we find no clear impact on the difference in mechanical properties of the matrix.

In summary, our findings showed a consistent impact of coculture with bone cells on PCa cells, in terms of both cell morphology and gene expression. These effects were observed regardless of the stiffness of the cancer ECM, implying that the interaction and communication with bone cells are a more significant factor in PCa progression than changes in cancer ECM stiffness.

### 2.4 Cell Morphologies and Gene Expression of Bone Cells in **S-PIC** of *TempEasy*

**Figure 4:**
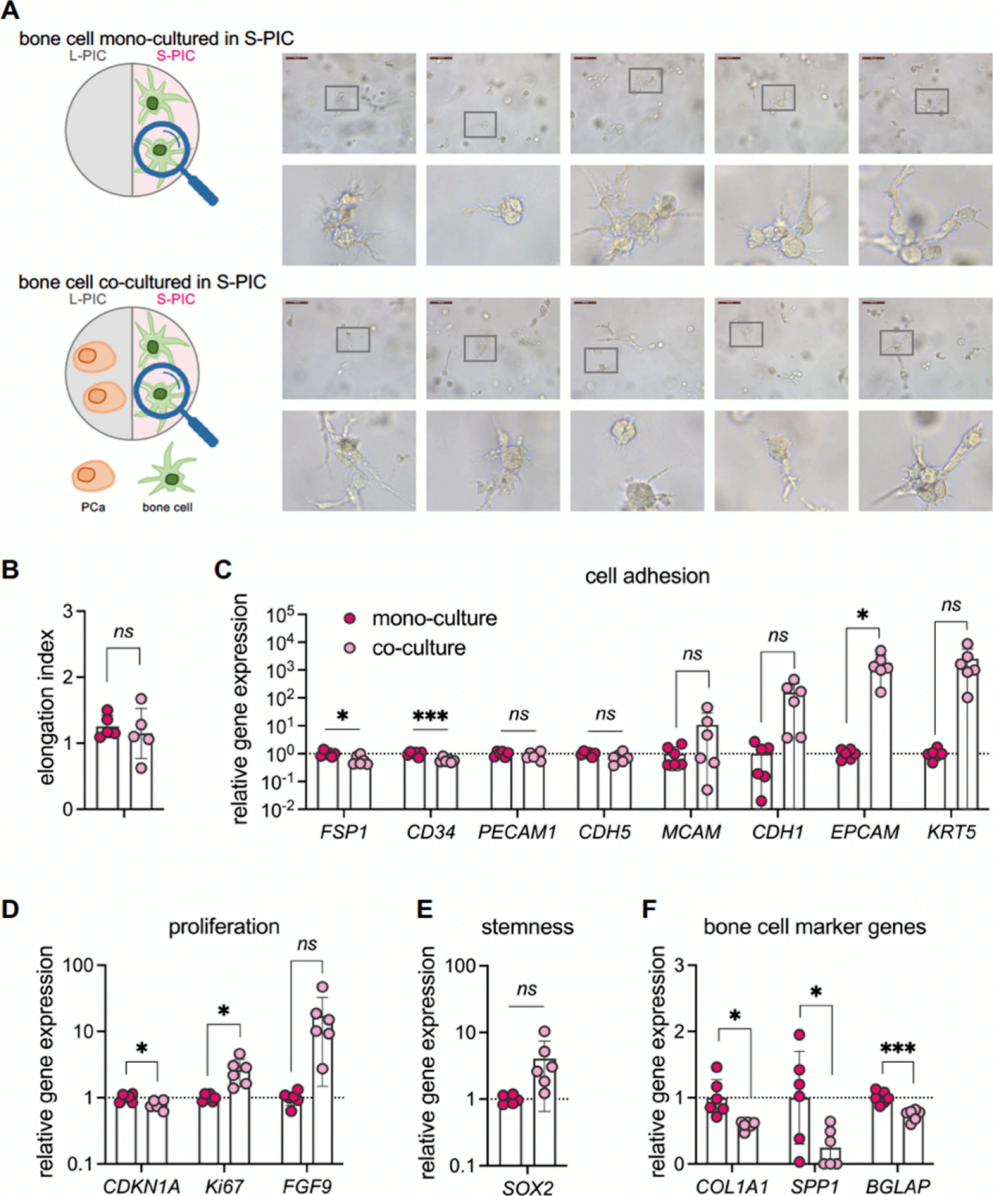
Bone cells in **S-PIC** of *TempEasy*. (A) Schematics of our experimental setup. Brightfield microscopy images of bone cells after 7 days in **S-PIC** hydrogel culture, either monoculture (top panel) or coculture with PCa cells (bottom panel). Concentrations, cell densities, and other conditions are given in the Materials and Methods section. (B) Quantification of elongation index of bone cells. (C–F) RT-qPCR quantification of marker gene expression, including cell adhesion makers (C), cell proliferation (D), stemness (E), and well-established bone cell markers (F), by comparing monoculture to coculture with bone cells. Results are expressed as mean ± SD of from six biological replicates. Statistical significance was determined using an unpaired *t* test with Welch’s correction: *ns* = not significant (*p* >0.05), *p <0.05, **p <0.01, ****p <0.0001.

To gain a deeper understanding of reverse paracrine effects during PCa bone metastasis, we evaluated changes in bone cells when cocultured with PCa cells. We compared bone cells grown in **S-PIC** hydrogel in monoculture and in coculture with PCa cells (Figure 4A). After 7 days in monoculture, the bone cells exhibited a rounded shape with protrusions stretching out, sometimes resulting in clustering (Figure 4A). However, when cocultured with PCa cells, the bone cell morphology remained largely unchanged. There was no significant difference in elongation factor measured between the monoculture and coculture with PCa cells (Figure 4B).

In addition to morphological changes, we examined gene expression in bone cells through a partially overlapping gene set (Figure 4C–F), including cell adhesion genes (*FSP1*, *CD34*, *PECAM1*, *CDH5*, *MCAM*, *CDH1*, *EPCAM*, and *KRT5*), cell proliferation markers (*CDKN1A*, *Ki67*, and *FGF9*), stemness marker (*SOX2*), and well-established bone cell markers (*COL1A1*, *SPP1*, and *BGLAP*).

Regarding cell adhesion markers, we noted a marked decrease in *FSP1* and *CD34* expression in bone cells when cocultured with PCa cells (Figure 4C), contrasting the significant upregulation of those genes in PCa cells upon coculture (Figure 2C). Moreover, we noticed a significant increase in *EPCAM1* expression up to 1000-fold. *EPCAM* (Epithelial Cell Adhesion Molecule) was initially identified as a tumor antigen in colorectal carcinomas and serves as a prognostic marker for disseminated tumor cells, which are considered the major source for metastatic cancer cells.^63^ Its high expression in bone cells has been reported rarely. Similarly, we observed upregulated expression of *CDH1* and *KRT5* (keratin 5), although the difference was not significant between monoculture and coculture. A recent bioinformatic study linked four key genes (*KRT5*, *HIPK2*, *MAP3K5*, and *CD5*) to osteosarcoma patient survival, with *KRT5* expression positively correlated with survival risk.^64^ Other cell adhesion markers (*PECAM1*, *CDH5*, and *MCAM*) showed no clear changes upon coculture with PCa cells (Figure 4C). Comparing bone cells (Figure 4C) to PCa cells (Figure 2C), we observed different sets of cell adhesion markers being differentially regulated, indicating cell-specific responses.

The negative cell cycle marker *CDKN1A* showed a minor however significant decrease (Figure 4D), indicating increased bone cell proliferation with coculture of PCa cells. To further confirm these results, we examined *Ki67* and *FGF9*, both cell proliferation markers. *Ki67* showed a significantly higher expression level, implying active dividing of bone cells, as *Ki67* levels are highest in the G_2_ phase and mitosis.^65^*FGF9* (Fibroblast Growth Factor 9), which promotes stem cell proliferation through p38 MAPK signaling,^66^ showed an increase in expression in bone cells with coculture, but the difference was not significant due to large variations (Figure 4D). Together, these markers suggest increased bone cell proliferation with coculture of PCa cells.

Unexpectedly, we observed higher expression of stemness marker *SOX2* after 7 days of coculture with PCa cells, indicating a less differentiated state (Figure 4E).^67^ We then tested three bone matrix protein genes, *COL1A1* (Collagen type I alpha 1), *SPP1* (Osteopontin), and *BGLAP* (Osteocalcin),^68^ whose gene expression was consistently lower in bone cells after coculture with PCa cells (Figure 4F). The decreased expression of bone matrix marker genes seems to contradict previous research showing *COL1A1* promoting breast cancer metastasis and SPP1 is involved in distant metastasis.^69−71^ This discrepancy, however, could originate from the short 7-day coculture, which does not fully mimic late-stage PCa bone metastasis. Together, our findings of the increased stemness and decreased bone matrix protein expression suggested that bone cells might undergo cell identity remodeling when cocultured with PCa cells, which may function as an important step during PCa progression.

### 2.5 Cell Morphologies and Gene Expression of Bone Cells in **L-PIC** of *TempEasy*

**Figure 5:**
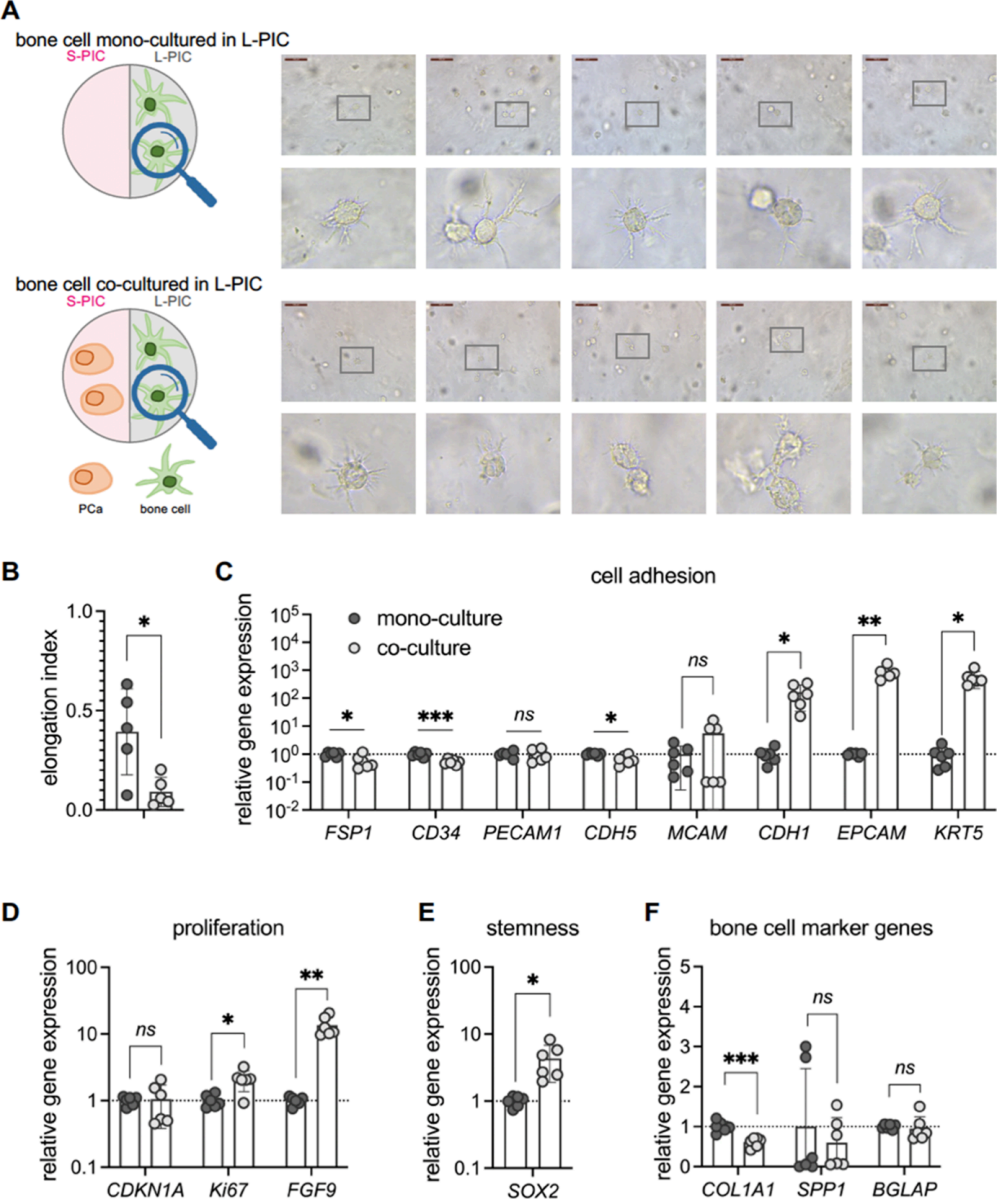
Bone cells in **L-PIC** of *TempEasy*. (A) Schematics of our experimental setup. Brightfield microscopy images of bone cells after 7 days in **L-PIC** hydrogel culture, either monoculture (top panel) or coculture with PCa cells (bottom panel). Concentrations, cell densities, and other conditions are given in the Materials and Methods section. (B) Quantification of the elongation index of bone cells. (C–F) RT-qPCR quantification of marker gene expression, including cell adhesion makers (C), cell proliferation (D), stemness (E), and well-established bone cell markers (F), by comparing monoculture to coculture with bone cells. Results are expressed as mean ± SD of from six biological replicates. Statistical significance was determined using an unpaired *t* test with Welch’s correction: ns = not significant (*p* >0.05), *p <0.05, **p <0.01, ****p <0.0001.

The most common site of metastases in patients with advanced PCa is the skeleton.^4,5,72^ Apart from the interactions between tumor cells and bone cells, previous studies indicated that bone metastases are also influenced by the bone microenvironment.^73^ After accessing bone cells in the **S-PIC** hydrogel compartment (Figure 4), we investigated if ECM stiffness changes affect cellular responses by culturing bone cells in the **L-PIC** hydrogel, followed by comparison between monoculture and coculture with PCa cells (Figure 5A). After 7 days, bone cells showed a rounded shape with protrusions stretching out in monoculture, whereas the protrusions are much shorter when cocultured with PCa cells (Figure 5A). In general, we barely observed any cell clustering of bone cells in the **L-PIC** hydrogel, which is different from when cultured in the **S-PIC** hydrogel (Figure 4A). As expected, the elongation factor is significantly smaller for bone cells in coculture with PCa cells (Figure 5B). We note that the observed morphological changes are not supported at the gene expression level, at least from the panel of genes we checked, both in monoculture and coculture conditions (Figures S4 and S5).

We next studied the effect of coculture with PCa cells on gene expression of bone cells in **L-PIC** hydrogel in the same four aspects as in **S-PIC** hydrogel (Figure 5C–F). First, with cell adhesion genes, we observed a significant downregulation of *FSP1*, *CD34*, and *CDH5* as well as a clear upregulation in the expression of *CDH1*, *EPCAM*, and *KRT5* (Figure 5C). This gene expression pattern is largely the same as what we observed in the **S-PIC** hydrogel (Figure 4C–F), with minor changes in statistics. Next, we evaluated cell proliferation by quantifying *CDKN1A*, *Ki67*, and *FGF9*. The higher expression of *Ki67* and *FGF9* indicated bone cells proliferate better when cocultured with PCa cells (Figure 5D). Lastly, we note a clear increase in *SOX2* expression and a significantly reduced *COL1A1* expression (Figure 5E,F).

Together, our findings showed a uniform effect of coculture with PCa cells on bone cells, especially regarding gene expression, independent of bone cell ECM stiffness (Figure 4 and 5, Figure S5), suggesting that interaction with PCa cells is a dominant influencing factor. Within a stiffer ECM, bone cells displayed morphological changes when cocultured with PCa cells (Figure 5B). Importantly, our results suggest that bone cells undergo cell identity remodeling when cocultured with PCa cells, with enhanced stemness and decreased bone matrix protein expression.

### 2.6 *In Vitro* Cell Staining

**Figure 6:**
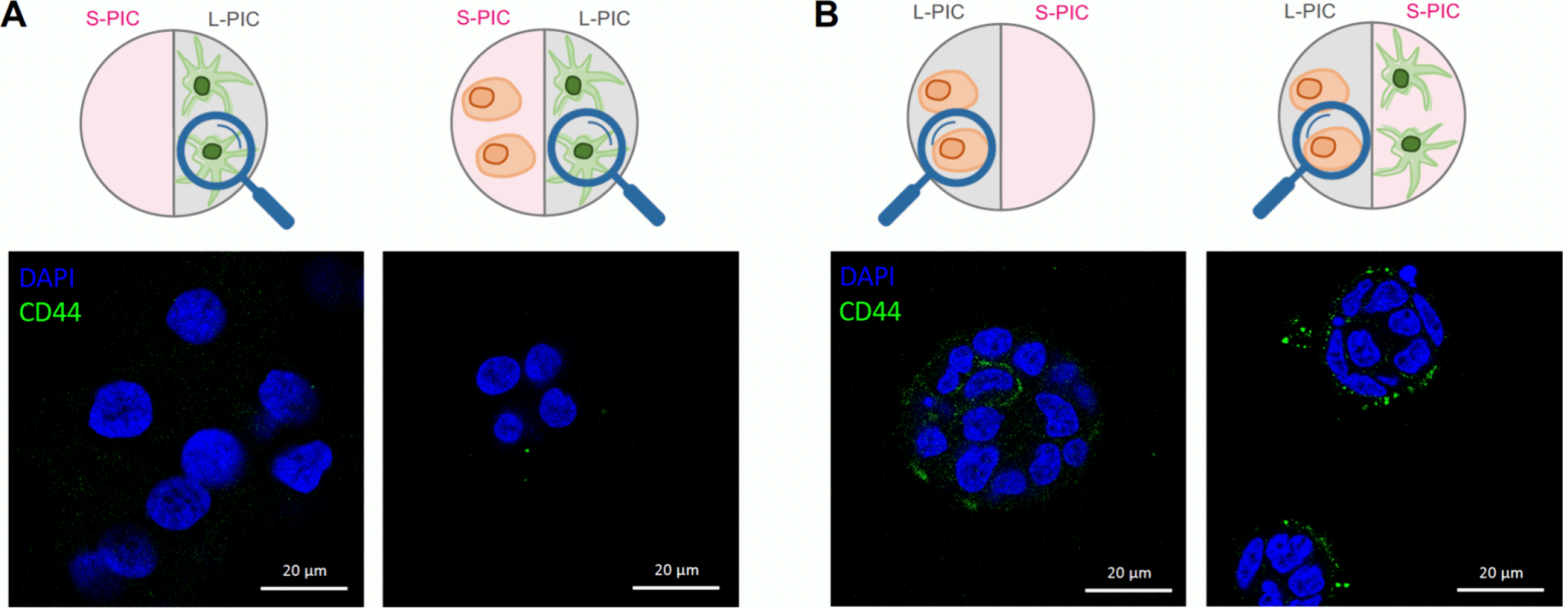
Immunofluorescence staining. CD44 (green) in bone cells (A) and in PCa cells (B) after 7-day monoculture or coculture. Nuclei are stained blue (DAPI). Scale bars: 20 μm.

An important concern about the *TempEasy* 3D coculture system is the potential mixing of cells between two compartments or incomplete removal of the S-PIC, which leads to a crossover of cells (and corresponding gene expression) in the wrong compartment. To exclude that these effects play a significant role, we validate the coculture system by immunofluorescence staining of CD44, which is widely acknowledged as a molecular marker for cancer stem cells and plays a pivotal role in communication with the microenvironment.^74^ The experiment focused on the boundary between the two compartments, where crossover may be expected. We observed that bone cells are depleted of CD44, while there is a clear enrichment of CD44 at the surface of PCa cells (Figure 6A,B). Note that the DAPI counterstaining clearly visualizes the spherical shape of PCa cell clusters (Figure 6B), which are not expected to metastasize to the other compartment, where bone cells are colonized in the short 7-day incubation period. We note that staining in the stiffer **L-PIC** compartment is experimentally easier, as the **S-PIC** gels are more sensitive toward the repeated washing steps in immunostaining protocols. Selective staining in **S-PIC** may require the use of a dedicated bis-DBCO cross-linker.^75^

## 3 Discussion

One of the persistent challenges in prostate cancer (PCa) research pertains to the insufficiency of suitable experimental models. The *TempEasy* system presents an advantageous framework to examine the interplay between PCa cells and bone cells, along with the concomitant influence of their respective extracellular matrix (ECM). Key advantages of *TempEasy* are the well-controlled cell extraction from the gels as well as the small-scale experimental setup, which allows for screening strategies. In this respect, straightforward RT-qPCR is an excellent tool to generate a vast amount of gene expression cell data. Staining experiments, while possible, will typically generate more qualitative results. The small-scale setup is less compatible with traditional protein analysis through Western Blotting, which would often require the pooling of wells. We note that, in the setup, the diameter of the wells may play an important role, as in larger wells, a larger fraction of cells will be further away from the interface and may experience different paracrine signal concentrations or gradients.

**Figure 7:**
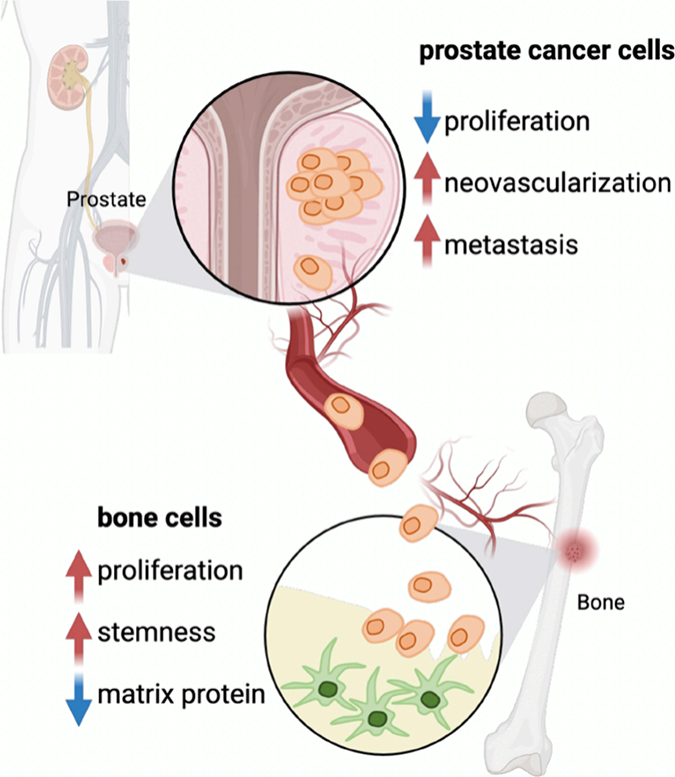
PCa bone metastasis model. Our results showed that, after 7 days of coculture, there was a decrease in cell proliferation but an increase in neovascularization as well as the metastatic potential of PCa cells. On the other hand, bone cells exhibited an increase in cell proliferation, stemness, and a decrease in bone matrix protein expression. Image created with BioRender.com.

Our findings revealed a discernible reduction in cell proliferation of PCa cells, with an increase in their metastatic potential (Figure 7), following a seven-day indirect coculture with bone cells. Conversely, bone cells exhibit an escalated rate of cell proliferation and increased stemness while displaying lower expression of bone matrix proteins after a seven-day coculture with PCa cells. The coculture effects are consistent in both soft **S-PIC** and stiff **L-PIC** hydrogel microenvironments, which indicates that the stiffness of the ECM is unlikely to exert a prominent role in the context of PCa bone metastasis in our current experimental setup. Together, our findings offer primary, albeit valuable, molecular insights into the intricacies of site-specific lodging of PCa cells within the bone microenvironment.

In this study, we observed highly consistent molecular responses within PCa cells following a coculture with bone cells (Figures 2 and 3, Figure S3). Among the panel of examined genes, a cohort of five genes displayed uniform upregulation in both soft **S-PIC** and rigid **L-PIC** environments. These genes, namely, *PECAM*, *CDKN1A*, *FGFR1*, *VEGFA*, and *IL8*, collectively signify a reduced cellular proliferation rate and augmented potential for neovascularization and, consequently, metastasis. First, the increased expression of *CDKN1A* provides initial evidence for suppressed PCa cell proliferation, given its documented inverse correlation with PCa growth both *in vitro* and *in vivo*.^51−53^ Second, the remaining four genes have been previously associated with early angiogenic events, underscoring their pivotal roles in tumor vascularization. Angiogenesis, the process of generating new blood vessels, is crucial for tumor development and progression, facilitating the provision of oxygen and nutrients to the expanding neoplasm. *PECAM*, for instance, has been linked with early angiogenic transitions, serving as an indicator for the recruitment of new vasculature in high-grade prostatic epithelial neoplasia.^43^ In the context of PCa, FGFR1-mediated epithelial-stromal interactions have been implicated in pathogenesis, as evidenced by experiments involving genetically engineered mouse models.^76^ Notably, clinical samples have also revealed aberrant expression patterns of FGFR isoforms during human PCa progression.^77^ The proangiogenic factors VEGFA, VEGFB, and VEGFC have been identified as pivotal components in various tumor types, with the expression of VEGF observed across normal, benign, and malignant prostate cells.^58,78−81^ IL-8, another proangiogenic factor,^82^ exhibited significant upregulation in our *TempEasy* system, consistent with findings from other 3D model investigations.^56^

The upregulation of *FSP1* and *CD34* was exclusively observed in PCa cells cultured within the stiff **L-PIC** environment. FSP1 enhances tumorigenesis and subsequent invasion of human prostate cancer by regulating the transcription of matrix metalloproteinase 9.^45,46^ CD34 serves as an endothelial cell marker, with previous investigations illustrating its association with enhanced tumor progression and aggressiveness in prostate cancer.^47^ More particularly, CD34 expression is associated with newly formed vascular endothelium, and heightened CD34 expression in tumor tissue signifies pronounced tumor neovascularization.^48^ These findings align with our observation of the increased neovascularization signature at the molecular level in PCa cells during the coculture with bone cells. The specific upregulation of *FSP1* and *CD34* within the context of the rigid **L-PIC** gel matrix suggests a potential sensitivity of these genes to ECM stiffness, although further validation is desired. To sum up, the *TempEasy* coculture systems reproduce key hallmarks of the initial stages of the angiogenic transition in prostate cancer, a phenomenon challenging to discern due to its occurrence before the definitive clinical diagnosis.

On the other hand, comprehensive characterization of bone cells in the context of prostate cancer, particularly concerning molecular alterations, has remained limited in the literature. We evaluated molecular changes across several aspects including cell adhesion, cell proliferation, stemness, and bone cell identity markers. Among them, *EPCAM* and *Ki67* were upregulated while *FSP1*, *CD34*, and *COL1A1* exhibited significant downregulation in bone cells following a seven-day coculture with PCa cells (Figures 4 and 5, Figure S5). This observation is consistent in both soft **S-PIC** and rigid **L-PIC** environments. EPCAM was discovered four decades ago as a tumor antigen in colorectal carcinomas and serves as an anchor molecule on circulating tumor cells (CTCs), which are considered the major source of metastatic cancer cells.^83^ Recent data indicate that EPCAM becomes downregulated by approximately 10-fold on cancer cells during dissemination into the bloodstream.^84^ Another transcriptome profiling study in colorectal cancer validated EPCAM downregulation on CTCs compared to primary tumors.^85^ However, a limited literature is available regarding EPCAM expression patterns in bone cells. We postulate that the increased level of expression of EPCAM in bone cells might potentially facilitate PCa progression, although this hypothesis necessitates thorough validation.

In contrast, it is rather clear that the increased expression of *Ki67* indicates an increase in cell proliferation of bone cells, as *Ki67* levels are highest in the G_2_ phase and mitosis.^65^ Interpretation of the diminished expression of *FSP1* and *CD34* remains challenging due to the lack of pertinent literature. FSP1 is only detectable in cells of the bone marrow, spleen, thymus, and lymphocytes.^45^ Both FSP1 and CD34 are associated with enhanced tumor progression;^45−47^ however, their role in bone cells has been scarcely reported.

Furthermore, COL1A1, a well-established bone matrix protein, exhibited downregulation, while prior research has indicated that expression of COL1A1 could be involved in promotion of breast cancer metastasis.^69^ This discrepancy may signify cancer-type-specific responses of bone cells. Notably, when cultured in **L-PIC**, the expression of SOX2 is substantially upregulated in bone cells, implying an increase in stemness. The SOX2 overexpression is common in many human cancers and is involved in various aspects of metastasis and unfavorably impacts drug resistance, leading to poor survival rates in cancer patients,^54,55^ whether these principles extend to bone cells necessitates further investigation. Based on our current findings, the reduced expression of cell identity markers and the increased expression of stemness markers together suggest that bone cells might be undergoing a transition to cellular identity.

In addition to cell–cell interactions, the cancer microenvironment has been recently identified as a major factor influencing the metastatic spread of tumor cells,^61^ as well as treatment resistance of cancer to radiotherapy and chemotherapy.^86−92^ For instance, during human breast cancer development, a gradual increase in collagen deposition has been observed, with a progressive linearization and thickening of interstitial collagen.^62^ In 3D coculture investigations about prostate cancer, the predominant focus, thus far, has been on the establishment of *in vitro* models.^17,20,34^ Consequently, the comprehensive exploration of the ECM’s role in prostate cancer has been relatively constrained, leading to its underappreciation when compared to its counterpart in different cancer types, notably breast cancer.^62,93^ To study whether perturbations within the ECM axis could influence the progression of prostate cancer, we have refined our *EasyTemp* coculture system by incorporating tunable matrix stiffness. Our investigation is centered on assessing the impact of ECM stiffness within a 3D milieu, both at the primary site involving PCa cells and at the distant malignant site involving bone cells. Over a 7-day coculture period, we have observed largely consistent alterations in both cell morphology and molecular dynamics within both the compliant **S-PIC** and rigid **L-PIC** hydrogel environments. These observations suggest that, within the current experimental setting, the stiffness of the ECM may not exert a predominant influence. However, it is imperative to exercise caution when interpreting these findings, given that a 7-day coculture duration is unlikely to accurately emulate the protracted *in vivo* metastatic process, which can span several years.^94^ Our findings are more indicative of the initial interaction phase, where ECM stiffness may not dominate the interactions between PCa and bone cells. To gain a more profound understanding of the impact of ECM stiffness in the later stages of PCa bone metastasis, more protracted coculture experiments are needed.

The primary site of metastases in advanced prostate cancer patients is bone tissue.^4,5,72^ Androgen ablation, a conventional therapeutic approach for prostate cancer, exacerbates osteoclastic bone resorption, leading to bone loss.^95,96^ Conversely, treatment with zoledronic acid has demonstrated the capacity to mitigate bone loss resulting from androgen deprivation and reduce bone metastases.^97,98^ As some patients may be more sensitive to bone loss than others, the selection of optimal treatment strategies should be contingent on individual patient circumstances. The utilization of patient-derived cell lines for further investigation holds promise for enhancing the comprehension of heterogeneous personalized responses and, consequently, refining individualized treatments. Our *TempEasy* system is poised to contribute to personalized medicine by serving as an *in vitro* model for drug response testing. However, a notable limitation of our current study lies in the use of only a single patient-derived cell culture. Using additional patient-derived organoid cultures in the future could offer a more representative depiction of tumor progression. Incorporating considerations of individual patient attributes, encompassing genetic mutations, age, and comorbidities, holds the potential to enhance our insights into PCa metastasis.

Acknowledging that tumor metastasis is an intricate and long-term process, characterized by complex molecular and cellular changes, as well as localized alterations like ECM and stromal remodeling, followed by systemic influences on the immune system,^99^ further investigations are warranted to elucidate the mechanisms underpinning PCa progression and metastasis. For instance, mechanical perturbations within solid tumors encompass solid stress and fluid pressure, which can detrimentally affect lymphatic drainage and blood vessel integrity.^100^

## 4 Conclusions

In summary, this work shows that the thermoresponsive character of the PIC gels can be exploited for 3D indirect coculture experiments. The true advantage of *TempEasy* is that the cells are easily extracted from their separate compartments and are available for downstream analysis. The RT-qPCR experiments we discuss in the metastasis study in this manuscript underline that this technique is suitable for relatively large-scale screening applications. In future endeavors, we intend to expand and refine our coculture system to better emulate the *in vivo* cancer microenvironment. Another avenue of exploration entails the incorporation of immune cells into our model system for tumorigenesis studies.

## 5 Materials and Methods

### 5.1 Polymer Synthesis and Peptide Conjugation

The synthesis of PIC polymers has been extensively described before.^37^ Azide (N_3_)-functionalized PIC polymers were synthesized through copolymerization of a methoxide and azide-appended monomer in toluene, using Ni (ClO_4_)_2_·6H_2_O as a catalyst. The resulting polymers, both **L-PIC** (low *T*_gel_) and **S-PIC** (high *T*_gel_), were obtained with a monomer:catalyst ratio of 1:5000 or 1:500. Viscosity-averaged molecular weights (*M*_v_) and from those polymer contour lengths (*L*_C_) were measured by viscometry.^35,36^ For biofunctionalization with the cell adhesive peptide GRGDS, a published protocol with the strain-promoted azide–alkyne cycloaddition reaction was used.^36^

### 5.2 Rheology Measurements

According to our previous *TempEasy* work,^23,101^ mechanical properties were assessed using a stress-controlled rheometer (Discovery HR-1, TA Instruments, steel parallel plate geometry with diameter: 40 mm, gap: 500 μm). Precooled samples were loaded onto the rheometer at 5 °C, and storage modulus *G*′ and loss modulus *G*″ were measured under oscillation (strain γ: 4%, frequency ω: 1.0 Hz) during a heating ramp to 37 °C (rate of 1 °C min^–1^). A frequency sweep was conducted at a strain of γ = 4%. Then, samples were cooled to 5 °C at the same rate. Each measurement was repeated three times for consistency.

### 5.3 Cell Culture and Encapsulation

MSK-PCa1 prostate cancer organoid cells were kindly provided by Dr. W.R. Karthaus (formerly Memorial Sloan Kettering Cancer Center, New York, USA), and organoids were cultured in the organoid medium.^102^ MG-63 bone cells were kindly provided by Dr. Frank Wagener from the Department of Dentistry, Radboud university medical center. MG-63 bone cells were cultured in Dubecco’s Modified Eagle Medium (DMEM) supplemented with 10% fetal bovine serum (Sigma-Aldrich, USA) and 1% penicillin/streptomycin (Gibco, Thermo Fisher, USA). Regular mycoplasma testing ensured no contamination. Both cell types were cultured at 37 °C in 5% CO_2_ in a humidified incubator with medium renewal every 2–3 days. Upon reaching 80–90% confluence, cells were trypsinized, centrifuged at 250 g for 5 min, and resuspended in fresh medium to a cell density of 2 × 10^5^/mL. Cell counts were performed using a LUNA-FL dual fluorescence cell counter.

### 5.4 Establishing the PCa and Bone Cell 3D Coculture Model

Dry **S-PIC** and **L-PIC** polymers underwent UV sterilization for 20 min before being dissolved in an ice-cold organoid medium (3 mg/mL) for 24 h at 4 °C prior to cell encapsulation. For encapsulation, the standard protocol from the *TempEasy* system was followed.^23^ Basically, cells (either PCa cells or bone cells) were mixed with the polymer solution on ice in a 1:1 ratio to reach a desired cell density of 1 × 10^5^/mL and a polymer concentration of 1.5 mg/mL. After thorough mixing, the **L-PIC**-cell mixture was kept on ice, while the S-S-PIC-cell mixture was maintained at 25 °C in a water bath to prevent a temperature drop below *T*_gel_. Next, 300 μL of **L-PIC**-cell mixture was pipetted with the 3D-printed mold placed in a 24-well plate well (Figure 1C) and incubated at 37 °C for 15 min to complete gel formation. Subsequently, the 3D-printed mold was removed, and 300 μL of **S-PIC**-cell mixture was pipetted next to the **L-PIC**-cell mixture. After another 15 min for gel formation, 300 μL of 37 °C preheated culture medium was dropwise added to the gel surface. All samples were then cultured under standard conditions (37 °C and 5% CO_2_).

Following 7-day coculture, the 24-well plate was taken out from the incubator, and 300 μL 15 °C precooled organoid medium was dropwise added to each well to retrieve the **S-PIC** encapsulated cells. This process was repeated twice. Subsequently, 300 μL of 4 °C precooled organoid medium was dropwise added to each well to collect the **L-PIC** encapsulated cells.

### 5.5 RNA Extraction, Reverse Transcription, and Quantitative Real-Time Reverse Transcription PCR (RT-qPCR)

These steps were performed according to our previous *TempEasy* study.^23^ Briefly, cells were washed twice with ice-cold PBS buffer and then pelleted at 250 g for 5 min. Subsequently, 0.5 mL of TRIzol Reagent (Invitrogen, Thermo Fisher, USA) was added to lyse the cells. After 5 min of incubation at room temperature, 0.1 mL of chloroform (Sigma-Aldrich, USA) was added, followed by vigorous vortexing and another 2 min incubation. Following centrifugation at 12,000*g* for 15 min, the upper aqueous phase was transferred and mixed with isopropyl alcohol (Sigma-Aldrich, USA) for RNA precipitation. After 30 min of incubation at −20 °C, RNA was collected by centrifugation at 12,000*g* for 15 min, followed by twice wash with 70% ethanol, air-dried, and finally dissolved in water.

Total RNA (500 ng) was used for DNase I (Invitrogen 18068-015, Thermo Fisher, USA) treatment. cDNA synthesis was performed using 200 U SuperScript II reverse transcriptase (Invitrogen 18064-014, Thermo Fisher, USA) and random hexamers. The resulting cDNA was stored at −20 °C.

RT-qPCR primers were designed with Primer3 (https://bioinfo.ut.ee/primer3-0.4.0/). Each primer set was first tested for the linear amplification dynamic range using a cDNA serial dilution. The primer sequences are listed in Table 1. RT-qPCRs were conducted using GoTaq qPCR (Promega) following the standard protocol. Human acidic ribosomal protein (*hARP*) serves as the housekeeping gene for normalization. Differences in gene expression were determined by the 2ΔΔ*C*_t_ method.^44^

**Table 1:** RT-qPCR Primers

primer	forward sequence	reverse sequence
BGLAP	CGCTACCTGTATCAATGGCTGG	CTCCTGAAAGCCGATGTGGTCA
CD34	CCTCAGTGTCTACTGCTGGTCT	GGAATAGCTCTGGTGGCTTGCA
CDH1	CGGGAATGCAGTTGAGGATC	AGGATGGTGTAAGCGATGGC
CDH5	GAAGCAGGCCAGGTATGAGA	CAAATGTGTACTTGGTCTGGGTG
CDKN1A	AAAAACTAGGCGGTTGAATGAGA	AAATAAAAATGCCCAGCACTCTTAG
COL1A1	GATTCCCTGGACCTAAAGGTGC	AGCCTCTCCATCTTTGCCAGCA
EPCAM	CCAGAACAATGATGGGCTTT	ACGCGTTGTGATCTCCTTCT
EZH2	GACCTCTGTCTTACTTGTGGAGC	CGTCAGATGGTGCCAGCAATAG
FGF9	ATGGCTCCCTTAGGTGAAGTT	CCCAGGTGGTCACTTAACAAAAC
FGFR1	GCACATCCAGTGGCTAAAGCAC	AGCACCTCCATCTCTTTGTCGG
FSP1	CTGGTACGGCGAGAGCAT	CAGGCTCCGGTGTGACTC
hARP	CACCATTGAAATCCTGAGTGATGT	TGACAAGCCCAAAGGAGAAG
IL8	GAGAGTGATTGAGAGTGGACCAC	CACAACCCTCTGCACCCAGTTT
Ki67	AAACCAACAAAGAGGAACACAAATT	GTCTGGAGCGCAGGGATATTC
KRT5	ATCTCTGAGATGAACCGGATGATC	CAGATTGGCGCACTGTTTCTT
MCAM	CTCTTCCTGGAGCTGGTCAAT	TTCGCTCTTACGAGACGGGG
NRP1	AACAACGGCTCGGACTGGAAGA	GGTAGATCCTGATGAATCGCGTG
PECAM1	GACGTGCAGTACACGGAAGT	GGAGCCTTCCGTTCTAGAGTAT
SOX2	CCCACCTACAGCATGTCCTACTC	TGGAGTGGGAGGAAGAGGTAAC
SPP1	CGAGGTGATAGTGTGGTTTATGG	GCACCATTCAACTCCTCGCTTTC
VEGFA	GAGATGAGCT TCCTACAGCAC	TCACCGCCTCGGCTTGTCACAT
VWF	AGTGGGATCTGCCAGTACCT	GATGCGGAGGTCACCTTTCA

### 5.6 Immunofluorescence Staining

For immunofluorescence analysis, 3D-cultured cells in PIC hydrogels were fixed with 3% paraformaldehyde (PFA) in PBS at room temperature for 30 min, followed by permeabilization with 0.5% (v/v) Triton X-100 in PBS for 20 min at 37 °C. Afterward, 3D-cultured cells in PIC hydrogels were carefully washed twice in PBS with 0.05% (v/v) Tween 20 for 3 min at 37 °C. Then, the samples were incubated in a blocking buffer (100 mM glycine) for 30 min at 37 °C. The first antibody, Anti-CD44 (1:800, HPA005785, Sigma-Aldrich, USA), incubation step was performed at 37 °C overnight in the blocking buffer. Subsequently, the samples were washed 3 times in PBS with 0.05% (v/v) Tween 20 for 3 min at 37 °C. The Alexa 488 conjugated secondary antibody (1:400, A-11008, Thermo Fisher, USA) incubation step was performed at 37 °C for 1 h in the blocking buffer. The samples were again washed 3 times in PBS with 0.05% (v/v) Tween 20 for 3 min, followed by a final wash in PBS at 37 °C. Then, 1:100 diluted DAPI (1 mg/mL) was added to samples at 37 °C. A quick rinse of PBS was performed after 10 min at 37 °C. Finally, PBS was added to each sample and kept at 37 °C. Immunofluorescence images were captured with a Leica DM6000 microscope (Leica) and processed using Fiji.

### 5.7 Statistical Analysis

Statistically analyses were conducted using the unpaired *t* test with Welch’s correction in the GraphPad Prism 5.0 software. Data are presented as mean ± SD, with sample sizes specified in the figure legends. *P-*Values indicating statistical levels are described as *ns* = not significant (*p* >0.05), *p <0.05, **p <0.01, ***p <0.001, ****p <0.0001.

## Supporting Information Available

Additional experimental results (PDF), mechanical analysis, and additional RT-qPCR results on mechanical effects in mono- and cocultures (PDF)
