# Supplementary material for: TempEasy 3D Hydrogel Coculture System Provides Mechanistic Insights into Prostate Cancer Bone Metastasis
Source: ACS Appl Mater Interfaces. 2024 May 13;16(20):25773–87. doi: 10.1021/acsami.4c03453 (PMC11129143; doi:10.1021/acsami.4c03453)
Supplement: Supplementary file 1 — am4c03453_si_001.pdf [file am4c03453_si_001.pdf]

Supplementary information

for

TempEasy 3D Hydrogel Co-culture System Provides Mechanistic Insights into Prostate Cancer Bone Metastasis

Zhaobao Zhang,[a] *Wen Chen,*[a] *Mingchen Sun,*[a] *Tilly Aalders,*[b] *Gerald W. Verhaegh,*[b] *Paul H. J. Kouwer*[a,] *

[a] Institute for Molecules and Materials, Radboud University, Heyendaalseweg 135, 6525 AJ Nijmegen, The Netherlands.

[b] Department of Urology, Radboud Institute for Molecular Life Sciences, Radboud university medical center, Geert Grooteplein Zuid 28, 6525 GA, Nijmegen, The Netherlands

* E-mail: p.kouwer@science.ru.nl

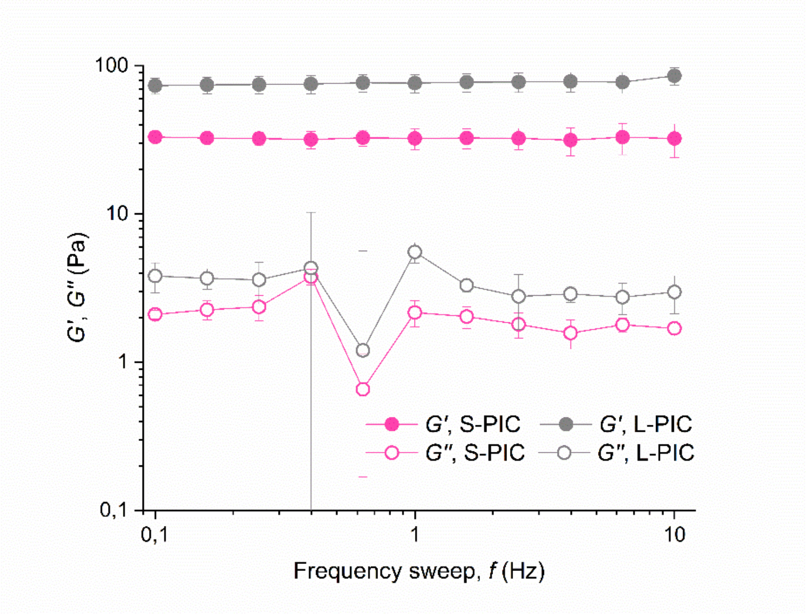

Figure S1. Viscoelastic properties: frequency sweep of gels as a function of PIC polymers with different length. The storage moduli *G′* and loss moduli *G″* at 37 °C show that the polymer length affects the mechanical properties of PIC hydrogels. Conditions: the concentrations of **S-PIC** and **L-PIC** are both 1.5 mg/mL.

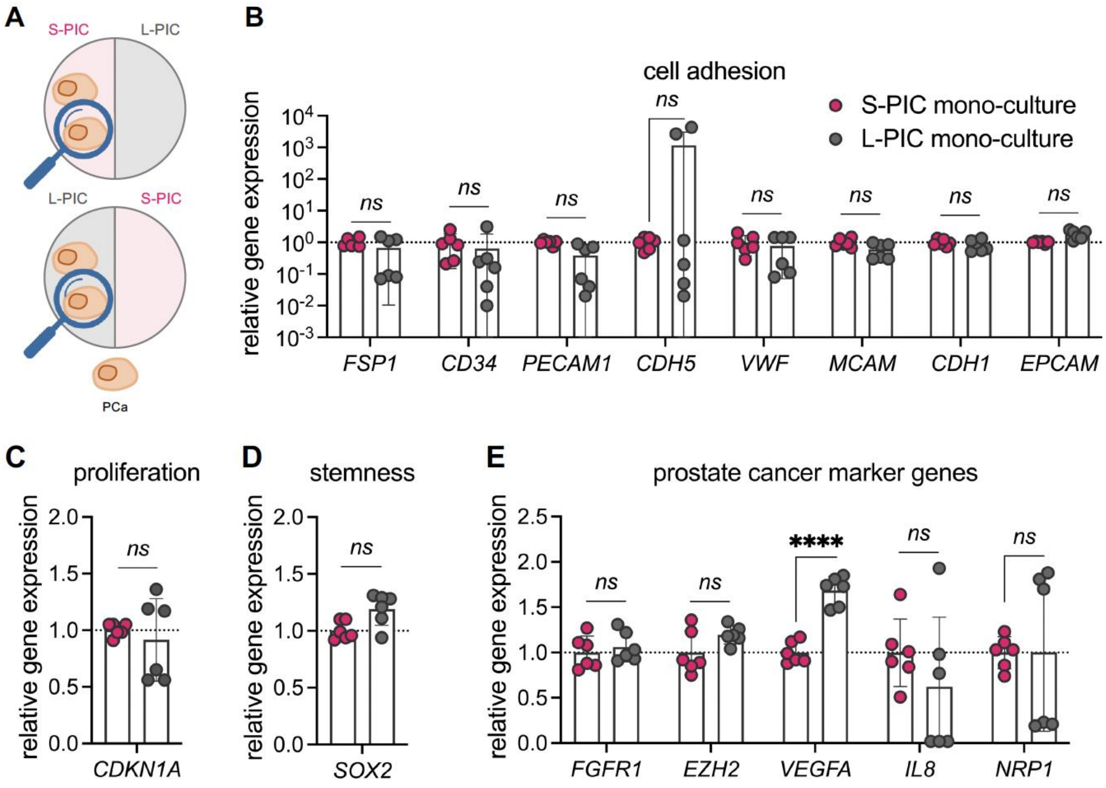

Figure S2. PCa cells in either S-PIC or L-PIC monoculture. (A) Schematics of our experimental setup. (B-E) RT-qPCR quantification of marker gene expression, including cell adhesion makers (B), cell proliferation (C), stemness (D), as well as reported PCa progression markers (E), by comparing monoculture to co-culture with bone cells. Data are shown as mean ± SD of n = 6 biological replicates. Statistics: *ns* = not significant ($p > 0.05$), **** $p < 0.0001$, by unpaired t-test with Welch's correction.

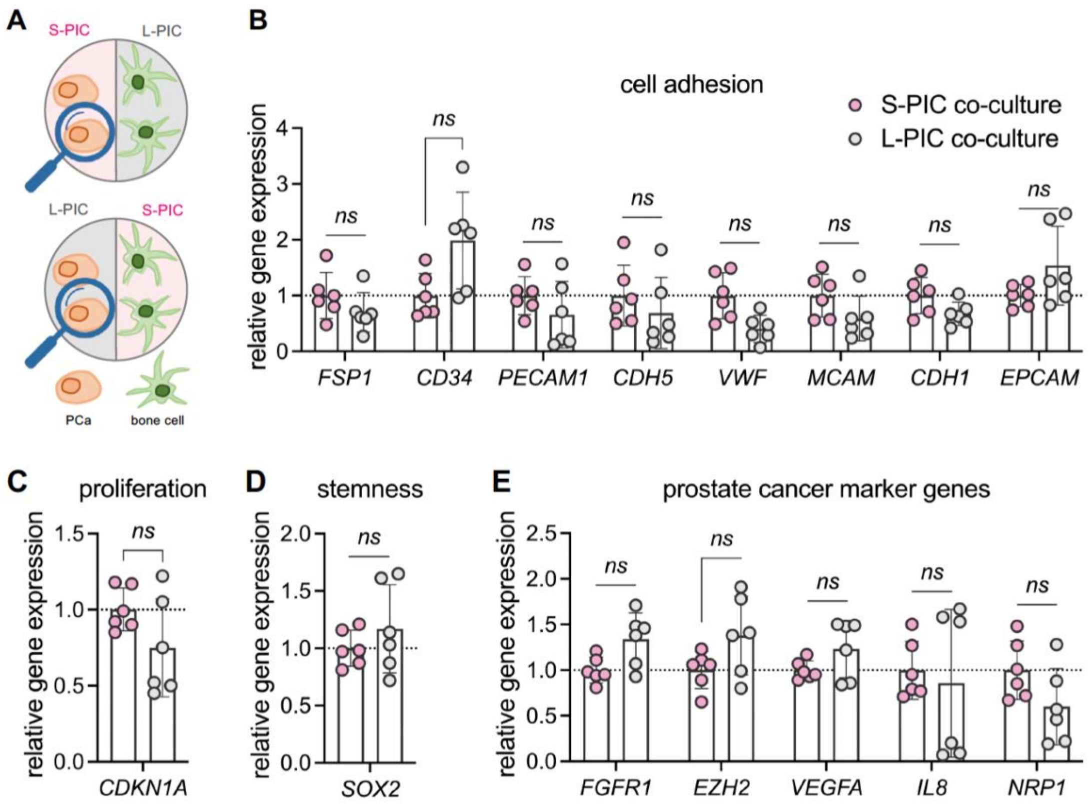

Figure S3. PCa cells in either S-PIC or L-PIC when co-cultured with bone cells. (A) Schematics of our experimental setup. (B-E) RT-qPCR quantification of marker gene expression, including cell adhesion makers (B), cell proliferation (C), stemness (D), as well as reported PCa progression markers (E), by comparing monoculture to co-culture with bone cells. Data are shown as mean ± SD of n = 6 biological replicates. Statistics: *ns* = not significant ($p > 0.05$), by unpaired t-test with Welch's correction.

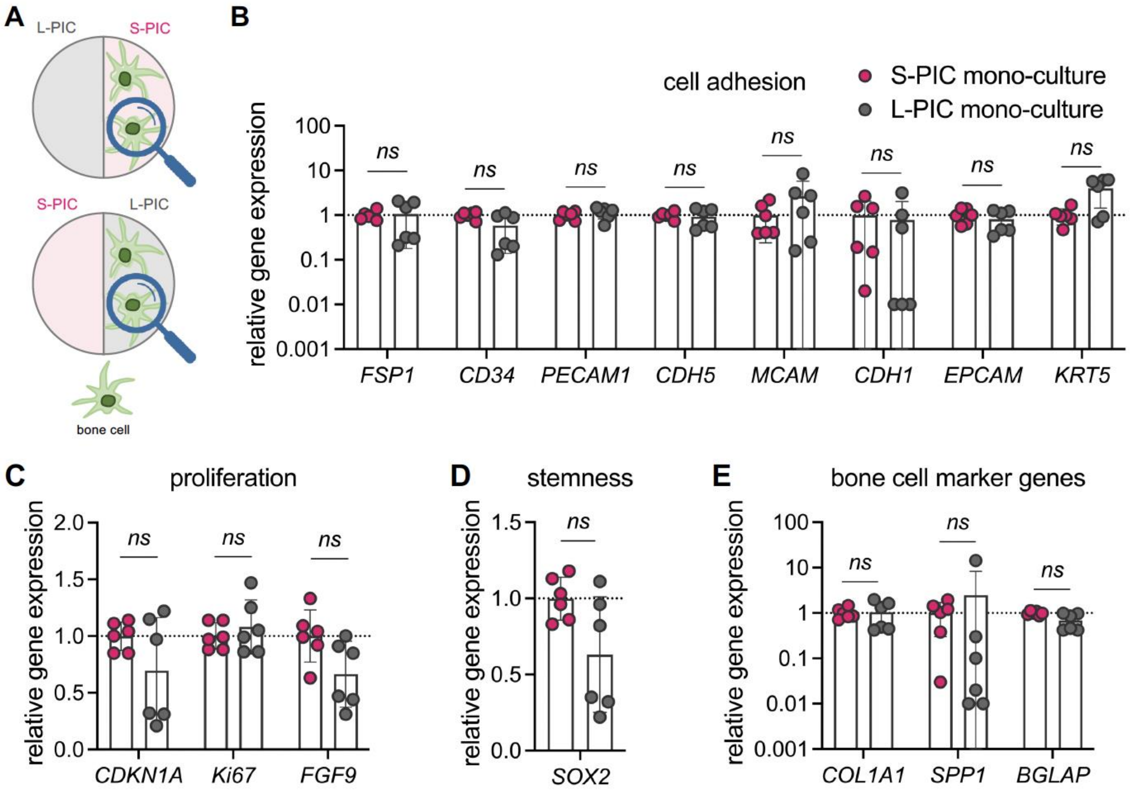

Figure S4. Bone cells in either S-PIC or L-PIC monoculture. (A) Schematics of our experimental setup. (B-E) RT-qPCR quantification of marker gene expression, including cell adhesion makers (B), cell proliferation (C), stemness (D), as well as well-established bone cell markers (E), by comparing monoculture to co-culture with bone cells. Data are shown as mean ± SD of n = 6 biological replicates. Statistics: *ns* = not significant ($p > 0.05$), by unpaired t-test with Welch's correction.

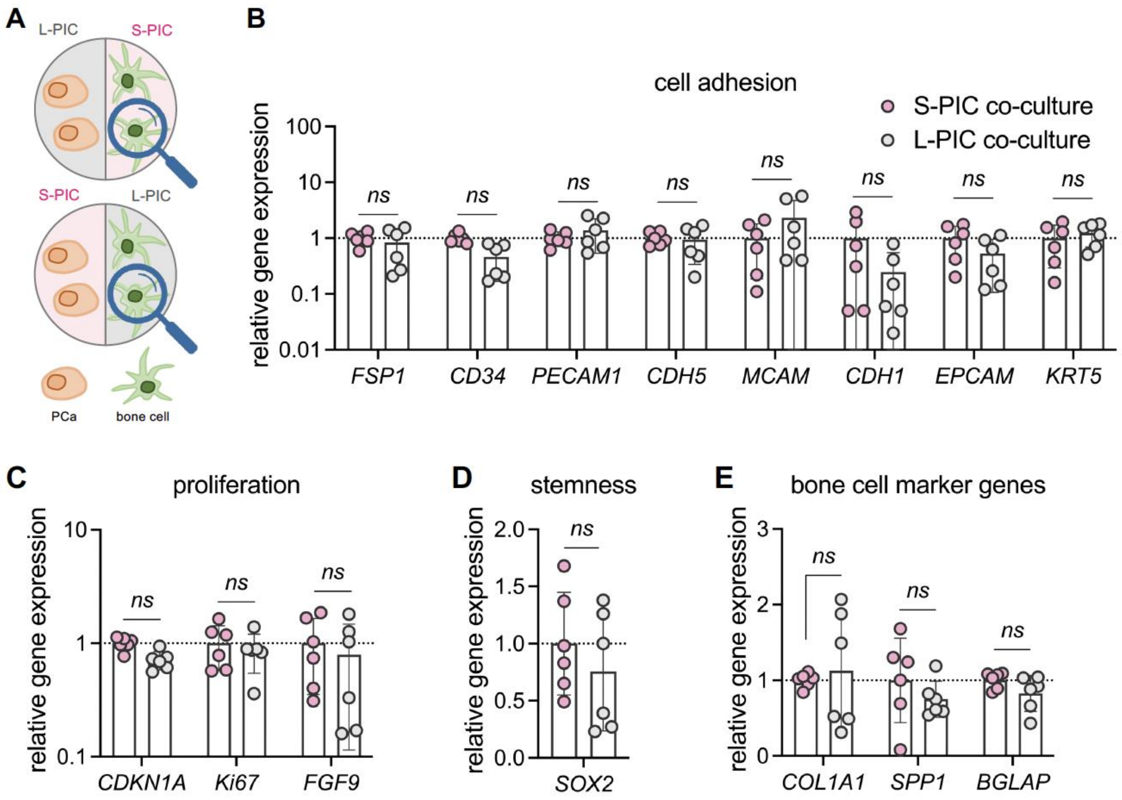

Figure S5. Bone cells in either S-PIC or L-PIC when co-cultured with PCa cells. (A) Schematics of our experimental setup. (B-E) RT-qPCR quantification of marker gene expression, including cell adhesion makers (B), cell proliferation (C), stemness (D), as well as well-established bone cell markers (E), by comparing monoculture to co-culture with bone cells. Data are shown as mean ± SD of n = 6 biological replicates. Statistics: *ns* = not significant ($p > 0.05$), by unpaired t-test with Welch's correction.
